# Search for Drugs Used in Hospitals to Treat Stomatitis

**DOI:** 10.3390/medicines6010019

**Published:** 2019-01-29

**Authors:** Yaeko Hara, Hiroshi Shiratuchi, Tadayoshi Kaneko, Hiroshi Sakagami

**Affiliations:** 1 Second Division of Oral and Maxillofacial Surgery, Department of Diagnostic and Therapeutic Sciences, Meikai University School of Dentistry, 1-1 Keyakidai, Sakado, Saitama 350-0283, Japan; 2 Department of Oral Maxillofacial Surgery, Nihon University School of Dentistry; 1-8-13 Kanda Surugadai, Chiyoda-ku, Tokyo 101-8310, Japan; shiratsuchi.hiroshi@nihon-u.ac.jp (H.S.); kaneko.tadayoshi@nihon-u.ac.jp (T.K.); 3 Meikai University Research Institute of Odontology (M-RIO), 1-1 Keyakidai, Sakado, Saitama 350-0283, Japan; sakagami@dent.meikai.ac.jp

**Keywords:** Chinese herbal remedies, stomatitis

## Abstract

Stomatitis is an inflammatory disease of the oral mucosa, often accompanied by pain. Usually it is represented by aphthous stomatitis, for which treatment steroid ointment is commonly used. However, in the cases of refractory or recurrent stomatitis, traditional herbal medicines have been used with favorable therapeutic effects. Chemotherapy, especially in the head and neck region, induces stomatitis at higher frequency, which directly affects the patient’s quality of life and treatment schedule. However, effective treatment for stomatitis has yet to be established. This article presents the clinical report of Kampo medicines on the stomatitis patients in the Nihon university, and then reviews the literature of traditional medicines for the treatment of stomatitis. Among eighteen Kampo medicines, Hangeshashinto has been the most popular for the treatment of stomatitis, due to its prominent anti-inflammatory activity. It was unexpected that clinical data of Hangeshashinto on stomatitis from Chinese hospital are not available. Kampo medicines have been most exclusively administered to elder person, as compared to pediatric population. Supplementation of alkaline plant extracts rich in lignin-carbohydrate complex may further extend the applicability of Kampo medicines to viral diseases.

## 1. Introduction

Stomatitis is an inflammation induced by various factors such as trauma, viruses and bacterial infections, genetic factors, stress and vitamin deficiency [1,2,3]. Chemotherapy and radiotherapy may produce active oxygen species and free radicals, that cause oxidative injury, inflammation of the oral mucosa and pain [4,5]. Like the digestive tract, oral mucosa membrane is susceptible to stress, and prone to be deteriorated by contact with teeth and unsanitary oral hygiene. Therefore, anti-stomatitis therapy with herbal medicines should be based on their anti-stress, anti-oxidative, mucous membrane protection and regeneration activities.

Western medicine usually consists of a single active ingredient and is prescribed to eradicate the causal diseases, based on the main complaint and examination data of the patients. In contrast, herbal medicines such as Japanese traditional medicine (Kampo) and Traditional Chinese Medicine (TCM) are mixtures of at least two kinds of constitutional plant extracts, are therefore applicable to various diseases [6]. ‘‘Oriental medicine’’ includes TCM, Korean medicine, Ayurveda (traditional Indian medicine) and Japanese Kampo medicine. TCM and medical texts were first brought to Japan from China during the 5–6th centuries. Until the 14–16th centuries, diagnosis and treatment were performed according to the theory of TCM, and thereafter developed, evolved and established independently in Japan, as a system of medicine that matches the environment and climate of Japan as well as the physical constitution and lifestyle of the Japanese population [6].

Currently, various clinical and fundamental studies have been conducted to elucidate the mechanism of the action of traditional medicines. This article presented the clinical report of Kampo medicines on the stomatitis patients in the Nihon university, and then reviewed the literature of traditional medicines for the treatment of stomatitis, based on the search by PubMed (National Center for Biotechnology Information, Bethesda, MD, USA) and Ichushi (Japan Medical Abstracts Society, Tokyo, Japan).

## 2. Kampo Medicines Prescribed in the Hospital of Nihon University School of Dentistry

**Figure 1:**
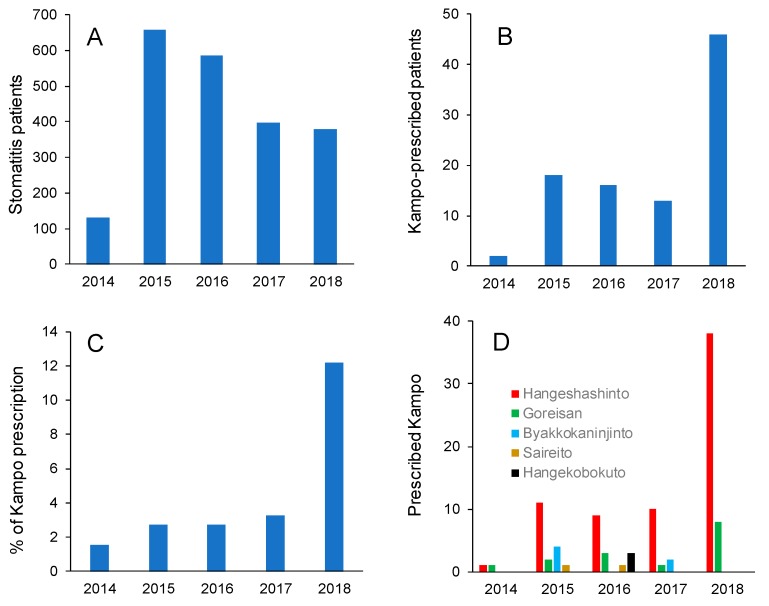
Changes in number of stomatitis patients (**A**), number of Kampo-prescribed stomatitis patients (**B**), percent of Kampo prescription (**C**) and number of prescribed Kampo medicines (**D**) during 2014 to 2018 (Data from hospital of Nihon University School of Dentistry).

We have surveyed approximately 400 patients with stomatitis in our hospital of the Nihon University School of Dentistry from January 2014 to October 2018. Number of patients with stomatitis progressively declined (Figure 1A), while the number of Kampo medicines prescribed to stomatitis patients was increased (Figure 1B). When the percent of Kampo medicines prescribed to stomatitis patient was calculated, it was found to be increased sharply in 2018 (Figure 1C). The most frequently prescribed Kampo medicine was Hangeshashinto (Figure 1D) (Figure 1).

**Figure 2:**
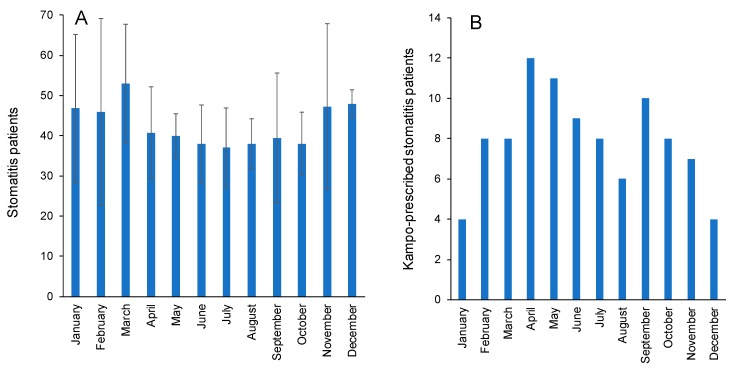
Number of stomatitis patients (**A**) and prescribed Kampo medicines in the hospital of Nihon University School of Dentistry (**B**).

During 12 months of years, the incidence of stomatitis was higher in winter season, peaked in March (Figure 2A), and the prescription of Kampo medicines peaked on April and May (Figure 2B), possibly to combat against the increasing numbers of stomatitis patients (Figure 2). 

Byakkokaninjinto, Goreisan, Hangekobokuto, Hangeshashinto and Saireito extract granules used in our hospital contains 5, 5, 5, 7 and 11 constituent plant extracts, respectively (Table 1). It should be noted that each extract contains numerous numbers of compounds.

During 5 years (2014–2018), 40 patients were treated with Hangeshashinto extract granules 2.5 g alone, while 27 patients were treated with hangeshashinto together with other Kampo medicines (Goreisan), gargle [sodium gualenate hydrate (azunol^®^ gargle liquid 4%), 0.2% benzethonium chloride solution (Neostelin Green 0.2% mouthwash solution)], anti-inflammatory agent (dexamethasone, triamicinolone acetonide, loxoprofen), or antimicrobial agent (miconazole gel) (Table 2). We first dissolve Hangeshashinto in water, and patients swallow it after gargling. When bitterness is too strong for the patients, we prescribe steroid ointment or azunol gargle in addition to Hangeshashinto, since azunol gargle protects the mucous membrane [7]. If mouth rinse is difficult, we will use other medicines.

**Figure 3:**
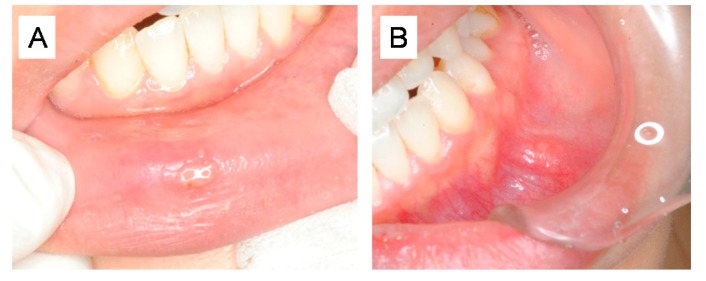
Therapeutic effect of Hangeshashinto on stomatitis. (**A**): Before Hangeshashinto treatment; (**B**): After Hangeshashinto treatment.

The following is the clinical report of stomatitis patients treated with Hangeshashinto in our hospital, after obtaining the informed consent from the patient, under the condition that the patient is not identified. The patient (female, 29 years old) was subjected to first medical examination on 23 May 2018. She showed the symptoms of stomatitis every few months. Each time, she applied triamcinolone acetonide (Kenalog®, Bristol-Myers Squibb Co., Tokyo, Japan) ointment herself, but got only short-term healing. When stomatitis developed again on early May 2018, Kenalog® did not work. Herpes simplex virus was detected in the oral cavity on 21 May. Administration of acyclovir, a popular anti-HSV agent, did not improved, but rather aggravated her symptom. Upon recommendation by the doctor, she got a close examination by the first author (Y.H.) on 23 May. The pain spread to the entire oral cavity, especially inside the anterior teeth part of the lower lip, and the tongue, feeling of incongruity during meals. There was no swelling or redness in the face. An ulcer suspected of stomatitis is formed in the buccal mucosa and the inner surface of the lips in the oral cavity. There was a tender pain with palpation (Figure 3A).

She was then treated with Hangeshashinto extract granules 2.5 g × 3 packages 14 days, and neostelin green mouthwash 0.2% 40 mL. On 6 June, a new stomatitis was formed in the molar part on the right upper side, and became slightly larger, however, the application of medicines was continued. It then became smaller and disappeared on 10 June. Some redness remained on the buccal gingiva of Upper right 6, but all other parts were healed. On 17 July, there was no mouth sores on the mucosal surface (Figure 3B).

## 3. Data Search for Traditional Medicine for the Treatment of Stomatitis

**Figure 4:**
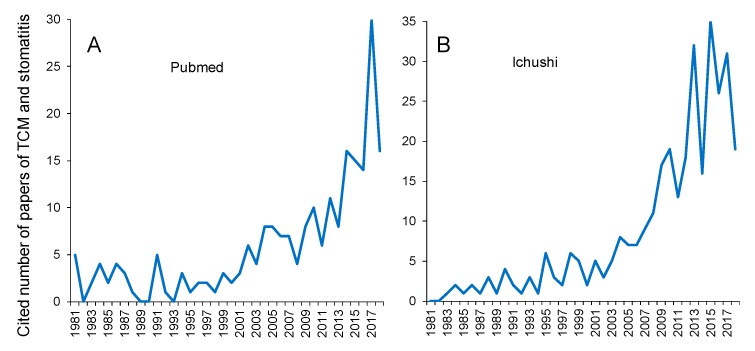
Increase of number of papers that cite TCM and stomatitis. (**A**): Data from Pubmed; (**B**): Data from Ichushi.

Stomatitis is a painful oral mucosal disorder, generated from various causes. Especially stomatitis in patients undergoing chemotherapy is severe, sometimes accompanied by eating difficulties. One common stomatitis often encountered is recurrent aphtha (recurrent aphthous stomatitis: RAS). RAS developed at a rate of 5–25% in the stomatitis patients [1], and treatment of RAS with Chinese patent medicines has been reported [8]. More recently, healing effects of Kampo on chemotherapy-induced stomatitis have been published [9,10]. Changes in the number of papers that related to Chinese Traditional Medicine was searched with PubMed (Figure 4A) and Ichushi (Figure 4B) (Figure 4). The publication of TCM appeared in 1980, and increased in the number more dramatically after 2000 in both cases. 

The most frequently used drugs for treatment of stomatitis, based on Pubmed search, were steroids (hydrocortisone acetate, triamcinolone acetonide, dexamethasone, beclometasone dipropionate (1 + 51 + 148 + 12 = 212 reports), followed by TCM (53 reports) > Kampo medicine (13 reports) and azunol ointment (main component: dimethyl isopropylazulene) (0 report) (Table 3). When corrected for the total numbers of references in each group, Kampo medicine was found to be the most popular for treating the stomatitis (0.92% of total application), followed by betamethasone (0.80%) > triamcinolone acetonide (0.74%) > beclometasone dipropionate (0.32%) > dexamethasone (0.22%) > hydrocortisone acetate (0.11%) > TCM (0.09%). It should be noted that Kampo medicine has been used for the purpose of treating stomatitis 10 times (= 0.92/0.09) than TCM (Table 3). 

**Figure 5:**
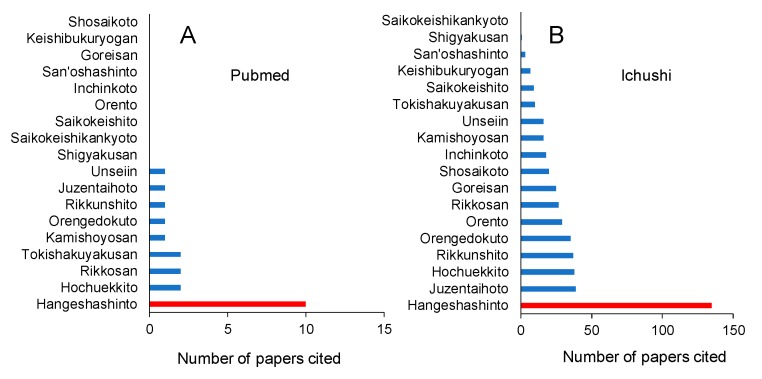
Hangeshashinto is the most popular kampo medicine for the treatment of stomatitis. Data obtained from Pubmed on 16 January 16 2019. (**A**): Data from pubmed; (**B**): Data from Ichushi.

A total of 18 Kampo medicines for the treatment of stomatitis are prescribed by hospitals and Rikkosan, Tokishakuyakusan> Kamishoyosan, Orengedokuto, Rikkunshito, Jpractitioners, according to the search with Pubmed and Ichushi. According to Pubmed, Hangeshashinto is the most frequently used [11,12], followed by Hochuekkito, uzentaihoto Unseiin > Shigyakusan, Saikokeishikankyoto, Saikokeishito, Orento, Inchinkoto, San’oshashinto, Goreisan, Keishibukuryogan and Shosaikoto (Figure 5A). The search by Ichushi reported the similar order of administration frequency: Hangeshashinto > Juzentaihoto, Hochuekkito > Rikkunshito > Orengedokuto > Orento > Rikkosan > Goreisan > Shosaikoto > Inchinkoto > Kamishoyosan > Unseiin > Tokishakuyakusan > Saikokeishito > Keishibukuryogan > San’oshashinto > Shigyakusan > Saikokeishikankyoto (Figure 5B).

Goreisan, known as “hydrostatic modulator” for edema, diarrhea, headache, nausea, and dizziness [13] is used to treat dry mouth. Rikkosan, a negative regulator of IL-1β network [14], plays a supplementary role for stomatitis by relieving the pain. Kamishoyosan (KSS), that enhances peripheral circulation and reduces stress and associated pain [15], and saikokeishikankyoto, that reduces posttraumatic stress [16], are effective to refractory and recurrent stomatitis. 

While hangeshashinto is mainly administered to patients with stomatitis at the middle to late stage, orento is used for the early stage of stomatitis, such as acute aphthous stomatitis [17]. This may be due to shorter treatment time of orento required for pain relief and complete cure (2.6 and 6.3 days, respectively), as compared with those of steroid ointment (7.5 and 12.3 days, respectively) [18]. Although orento is used clinically very often, the paper of orento is limited. This may be due to the fact that early stomatitis heals much faster, as compared to intractable, recurrent stomatitis and chemotherapy-induced stomatitis. Most of fundamental and clinical research studies have been focusing on the stomatitis in the patients with head and neck cancer who received chemotherapy or radiation chemotherapy, to keep the patient’s QOL and treatment continuity [19,20].

## 4. Application of Traditional Medicines for Pediatric Population

Kampo medicines and Hangeshashinto have been used for elderly person approximately one order higher rates, as compared with pediatric population (Table 4). Publication of TCM was approximately 44.5-fold (= 61,264/1409) as compared with Kampo medicine. Frequency of the use of TCM for elderly person was again one order higher than that for pediatrics (8494/844 = 10.1) (Table 4). This reflects that Kampo medicine and TCM are used to treat and improve the conditions of patients with many kinds of diseases.

These traditional medicines have been used to cure the skin diseases and abdominal pain, from long ago, although there are few reports on stomatitis for pediatric population. Licorice is a crude drug prescribed in various herbal formulas in traditional Japanese and Chinese medicines, and also used worldwide as a food natural sweetener [21]. Therefore, licorice makes it easier for medication use in children.

## 5. Why Hangeshashinto Is So Popular for the Treatment of Stomatitis?

**Figure 6:**
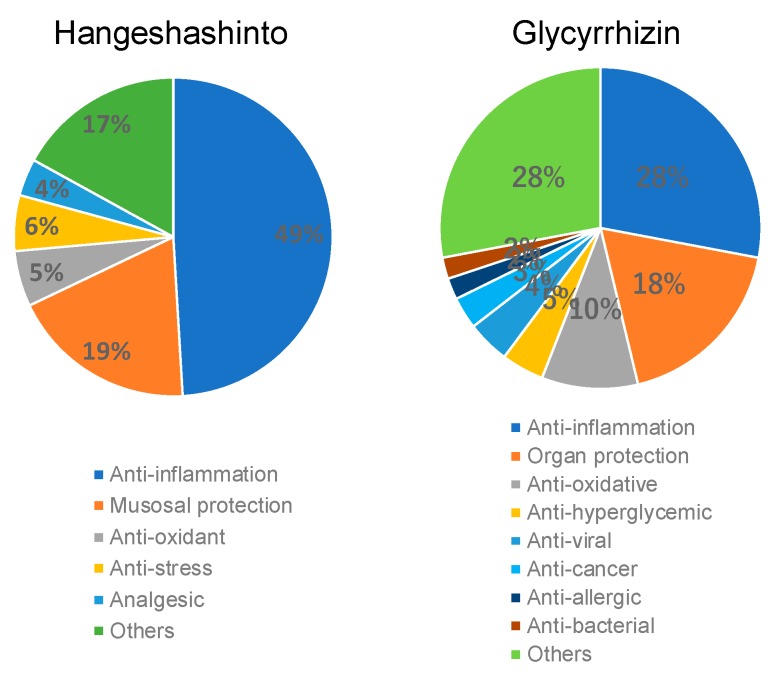
Prominent anti-inflammatory activity of Hangeshashinto.

Among seven constitutional plant extracts, glycyrrhiza and glycyrrhizin (the major component of glycyrrhiza) have been cited most frequently as the therapeutics for stomatis (Table 3). Among 53 papers that investigated the biological activity of Hangeshashinto, 26 papers (49%) dealt with its anti-inflammatory activity, followed by mucosal protection (ten papers, 19%), based on the search by PubMed. The most well-known biological activity of glycyrrhizin was again anti-inflammatory activity (Figure 6).

We have recently reported that Hangeshashinto and Glycyrrhiza inhibited PGE_2_ production in IL-1-β-stimulated human periodontal ligament fibroblast (selectivity index [SI (CC_50_/EC_50_) = 285 and 59, respectively) [22,23]. This suggests that anti-stomatitis activity of Hangeshashinto may be at least in part by Glycyrrhiza.

**Figure 7:**
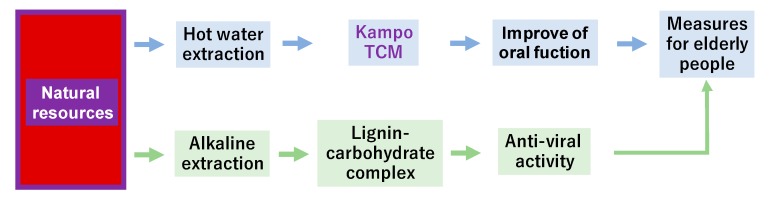
Supplementation of alkaline extract to Kampo prescription extends its therapeutic potential.

It has become increasingly apparent that oral health is co-related well with general health. Generally, the anti-HIV activity of Kampo medicine, prepared by hot water extraction, is generally weak [24]. However, alkaline extract of licorice extract [25], green tea, oolong tea and orange flower [26], that contain significant amount of lignin-carbohydrate complex, shows higher anti-viral activity than hot water extract. Supplementation of alkaline extract may further expand the therapeutic ranges of Kampo medicine (Figure 7).

## 6. Conclusions

Literature searches demonstrated that among 18 Kampo medicines, Hangeshashinto is most frequently used in Japan, possibly due to the presence of glycyrrhiza that contains anti-inflammatory glycyrrhizin. It was surprising that Hangeshashinto has not been used in China. Since Kampo medicines are prepared by hot water extraction, they have low levels of lignin–carbohydrate complexes (LCC) that are extracted by alkaline extracts and shows the prominent antiviral activity among three major polyphenols. By adding an alkaline extract rich in LCC to Kampo medicine, its therapeutic potential will become much broader. Up to now, traditional medicines have few cases of adaptation to children, but inclusion of sweet licorice ingredient will make it easier for children to take without resistance. Hangeshashinto is applicable for the treatment of stress gastritis, and seems to be the best Kampo medicine for the treatment of stomatitis, judging from the huge number of publications.

## Figures and Tables

**Table 1:** Kampo medicines and their constituent plant extracts used for treatment of stomatitis in the hospital of Nihon University School of Dentistry. BKTN, Byakkokaninjinto; GRS, Goreisan; HKT, Hangekobokuto; HST, Hangeshashinto; SRT, Saireito.

Constituent Plant Extracts	Kampo Medicines				
	BKNT	GRS	HKT	HST	SRT
Alisma Rhizome		〇			〇
Anemarrhena Rhizome	〇				
Astractylodes Lancea Rhizome		〇			〇
Brown Rice	〇				
Bupleurum Root					〇
Cinnamon Bark		〇			〇
Coptis Rhizome				〇	
Ginger			〇	〇	〇
Ginseng	〇			〇	〇
Glycyrrhiza	〇			〇	〇
Gypsum	〇				
Jujube				〇	〇
Magnolia Bark			〇		
Perilla Herb			〇		
Pinellia Tuber			〇	〇	〇
Polyporus Sclerotium		〇			
Poria Sclerotium		〇	〇		〇
Scutellaria Root				〇	〇

**Table 2:** Kampo medicines prescribed for stomatitis in the hospital of Nihon University School of Dentistry. All Kampo medicines are extract granules. BKTN, Byakkokaninjinto; BMZ, betamethasone; DX, dexamethasone, GRS, Goreisan; HKT, Hangekobokuto; HST, Hangeshashinto; SRT, Saireito; SC, Salcoat Capsule for oral spray; TAC, triamcinolone acetonide.

Kampo Medicine Prescribed	Number of Prescribed Kampo					
	2014	2015	2016	2017	2018	Total
HST 2.5 g/packet	0	8	5	9	18	40
HST 2.5 g/packet, SC 50 μg	0	1	0	0	0	1
GRS 2.5 g/packet	0	0	2	0	3	5
GRS 2.5 g + HST 2.5 g	0	0	1	0	0	1
BKTN 3 g + SC 50 μg	0	0	0	1	0	1
Azunol gargle 4% (10 mL) + HST 2.5 g	0	0	0	0	3	3
Azunol gargle 4% (10 mL) + HST 2.5 g + SC	0	0	0	0	2	2
Azunol gargle 4% (5 mL) + HST 2.5 g	0	1	2	0	0	3
TAC ointment 0.1% + Azunol + GRS 2.5 g	0	0	0	1	0	1
DX ointment 0.1% + SRT 3.0 g	0	1	0	0	0	1
DX oint 0.1% + HST 2.5 g	0	0	0	1	5	6
DX oint 0.1% + HST 2.5 g	0	0	1	0	0	1
DX oint 0.1% + GRS 2.5 g	1	0	0	0	3	4
DX oint 0.1%+ Azunol + HST 2.5 g	1	0	0	0	1	2
DX oint 0.1% + Neostelin Green gargle + HST 2.5 g	0	0	0	0	1	1
DX oint 0.1% + Neostelin + GRS 2.5 g	0	0	0	0	1	1
DX oint 0.1%+ Hachiazule gargle 0.1% + GRS 2.5 g	0	2	0	0	0	2
Neostelin green mouthwash + SRT 3 g	0	0	1	0	0	1
Neostelin green mouthwash + HST 2.5 g	0	0	0	0	3	3
Neostelin green mouthwash + GRS 2.5 g + SC 50 μg	0	0	0	0	1	1
Neostelin green mouthwash + BKTN 3 g	0	0	0	1	0	1
Miconazole gel 2% + HST 2.5 g	0	0	1	0	0	1
RACOL-NF Liquid for Enteral Use + HKT 2.5 g	0	0	1	0	0	1
BMZ/Gentamicin oint + Azonol 4% + HST 2.5 g	0	0	0	0	1	1
Loxoprofen tablet 60 mg + HST 2.5 g	0	1	0	0	0	1
Loxoprofen tablet 60 mg + HST 2.5 g + SC 50 μg	0	0	0	0	1	1
Hachiazule gargle 0.1% + BKTN 3 g	0	4	0	0	0	4
White petrolatum + Azunol 4% + HKT 2.5 g	0	0	1	0	0	1

**Table 3:** Medicines used for treatment of stomatitis (data obtained from Pubmed on 16 January 2019).

Medicines	Number of References		
	Alone	+ Stomatitis	%
	A	B	(A/B) × 100
Azunol Ointment (Dimethyl Isopropylazulene)	18	0	0
Hydrocortisone Acetate	890	1	0.11
Triamcinolone Acetonide	6891	51	0.74
Dexamethasone	68,125	148	0.22
Betamethasone	8478	68	0.80
Beclometasone	3751	12	0.32
Kampo Medicine	1406	13	0.92
Hangeshashinto	28	10	35.71
Coptis Rhizome	150	1	0.67
Ginger	3264	1	0.03
Ginseng	8868	4	0.05
Glycyrrhiza	3244	17	0.52
Glycyrrhizin	2389	7	0.29
Jujube	802	0	0
Pinellia Tuber	94	0	0
Scutellaria Root	502	2	0.40
Traditional Chinese medicine (TCM)	61,115	53	0.09

**Table 4:** Medicines used for treatment of stomatitis (data obtained from Pubmed on 16 January 2019).

Cited by	Number of References	% of Control
Kampo medicine (control)	1409	100.0
Kampo medicine + elderly	242	17.2
Kampo medicine + adult	287	20.0
Kampo medicine + young	43	3.1
Kampo medicine + child	33	2.3
Kampo medicine + pediatric	26	1.8
Hangeshashinto	28	100.0
Hangeshashinto + elderly	6	21.4
Hangeshashinto + adult	6	21.4
Hangeshashinto + young	1	3.6
Hangeshashinto + child	1	3.6
Hangeshashinto + pediatric	0	0.0
TCM	61,264	100.0
TCM + elderly	8494	13.9
TCM + adult	10,349	16.9
TCM + young	2343	3.8
TCM + child	1588	2.6
TCM + pediatric	844	1.4

## Abbreviations

BKTN	Byakkokaninjinto
BMZ	betamethasone
DX	dexamethasone
GRS	Goreisan
HIV	Human immunodeficiency virus
HKT	Hangekobokuto
HST	Hangeshashinto
LCC	Lignin-carbohydrate complex
SRT	Saireito
SC	Salcoat Capsule for oral spray
TAC	Triamcinolone Acetonide
TCM	Traditional Chinese Medicine
